# Biotin Availability Modulates Transglutaminase Expression and Activity during Lipid Accumulation in 3T3-F442A Adipocytes

**DOI:** 10.17912/micropub.biology.002186

**Published:** 2026-07-31

**Authors:** Cristina Velez-delValle, Federico Castro-Muñozledo, Walid Kuri-Harcuch, Teresita Padilla-Benavides

**Affiliations:** 1 Department of Cell Biology, Center for Research and Advanced Studies-IPN, Mexico City, Mexico; 2 Molecular Biology and Biochemistry, Wesleyan University, Middletown, Connecticut, United States

## Abstract

Biotin deficiency impairs lipid accumulation in 3T3-F442A adipocytes despite allowing adipocyte differentiation. Here, we examined whether transglutaminase, a cytoskeletal remodeling enzyme associated with lipid droplets, is affected by biotin availability. Under biotin-deficient conditions, adipocytes showed reduced lipid droplet formation, cytoplasmic empty vesicle-like structures, and decreased transglutaminase expression and activity. Pharmacological inhibition of transglutaminase reduced lipid accumulation and decreased malic enzyme activity, suggesting a link between transglutaminase activity and NADPH-dependent lipogenesis. These findings identify transglutaminase as a biotin-sensitive component associated with lipid accumulation in 3T3-F442A adipocytes.

**Figure 1. Biotin availability and transglutaminase activity regulate adipogenesis, lipid droplet formation, and lipogenic metabolism:** **(A)** Representative western blot and quantification of transglutaminase (TG2) expression during adipocyte differentiation under biotin-sufficient (B+) and biotin-deficient (B-) conditions. **(B)** Immunofluorescence analysis of perilipin (green) and TG2 (red) localization in differentiated adipocytes cultured in B+ and B- conditions. **(C)** Malic enzyme activity measured in total cell extracts from non-differentiated fibroblasts, cells one day prior to differentiation, and adipocytes differentiated for up to 7 days under B+, B-, and cadaverine-treated conditions. Data are presented as the mean ± SE from three independent biological replicates (N=3). *P < 0.05; **P < 0.01. Statistical analyses was performed with t-test to compare each time point to the adipocytes cultured with lipogenic media. **(D)** Representative bright-field and fluorescence of undifferentiated fibroblasts and adipocytes differentiated for 5 and 7 days in the presence or absence of cadaverine. BODIPY 488/503 was used to stain lipid droplets (N=3). **(E)** Quantification of BODIPY 488/503 fluorescence intensity shown in arbitrary units (a.u.). Statistical analysis was performed using one-way ANOVA followed by Bonferroni's multiple-comparisons test. Data is presented as the mean ± SE from three independent biological replicates (N=3). Lipogenic, non-lipogenic, cadaverine-treated, and undifferentiated fibroblast groups differed significantly, with comparisons yielding ***P < 0.001; ****P < 0.0001.

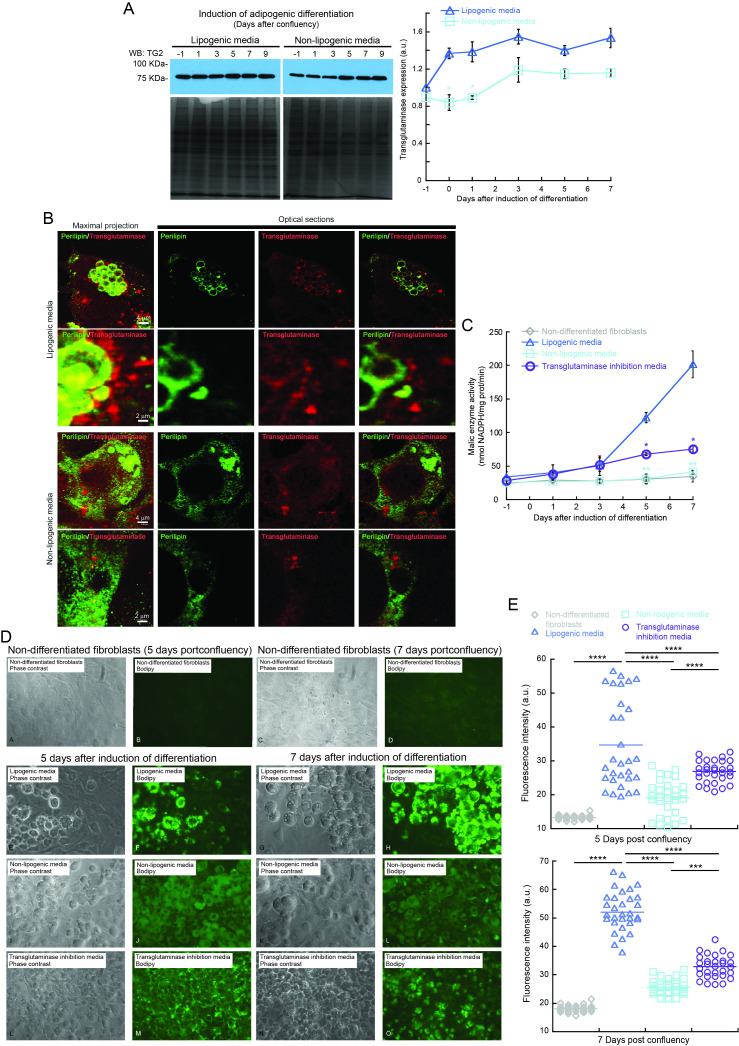



## Description

White adipocytes are specialized cells that store excess energy as neutral lipids, primarily triacylglycerols, within cytoplasmic lipid droplets. *In vivo* , white adipose tissue functions as the major site of energy storage and lipid mobilization, while also contributing to endocrine regulation through adipokine secretion (Luo and Liu, 2016). The murine 3T3-F442A cell line is a well-established model of white adipocyte differentiation and has been widely used to study the formation of lipid-laden adipocytes in culture (Green and Kehinde, 1976; Kuri-Harcuch et al., 1978). During differentiation, 3T3-F442A adipocytes undergo coordinated structural and metabolic remodeling that supports lipid droplet formation and expansion.

Lipid droplets arise in close association with the endoplasmic reticulum, are bounded by a phospholipid monolayer enriched in proteins such as perilipins, and interact with cytoskeletal elements, including vimentin intermediate filaments (Brasaemle et al., 1997; Brasaemle et al., 2004; Tauchi-Sato et al., 2002). During adipogenesis, small lipid droplets appear and progressively enlarge, ultimately forming the prominent lipid stores characteristic of mature white adipocytes (Green and Kehinde, 1976). Vimentin intermediate filaments undergo dynamic remodeling during this process and have been proposed to support lipid droplet organization and expansion (Franke et al., 1987; Novikoff et al., 1980; Lieber and Evans, 1996; Blanchette-Mackie et al., 1995).

The 3T3-F442A adipocyte model has been useful for distinguishing adipocyte differentiation from lipid accumulation. In an earlier study, culture in medium containing dialyzed serum prevented lipid accumulation, whereas addition of biotin restored lipid storage, indicating that the effect was attributable, at least in part, to removal of biotin from the serum by dialysis (Kuri-Harcuch et al., 1978). Under these biotin-deficient conditions, 3T3-F442A cells acquired several features of adipocyte differentiation but failed to accumulate normal lipid droplets. Ultrastructural analyses also revealed cytoplasmic vesicle-like structures in biotin-deficient cells, although their molecular identity and relationship to lipid droplet biogenesis were not defined at the time (Kuri-Harcuch et al., 1978). In that study, differentiation-associated enzymes behaved differently under biotin deficiency: glycerol-3-phosphate dehydrogenase, considered a primary adipogenic marker, was induced despite the absence of lipid accumulation, whereas malic enzyme, a secondary lipogenic enzyme associated with lipid accretion, was reduced (Kuri-Harcuch et al., 1978). Subsequent studies further suggested that lipid droplet formation in this model depends on coordinated interactions among cytoskeletal proteins, lipid droplet-associated proteins, and lipogenic enzymes (Padilla-Benavides et al., 2016).

Transglutaminase is a candidate mediator of this coordination because it catalyzes Ca ^2+^ -dependent transamidation reactions and participates in protein crosslinking, cytoskeletal remodeling, and extracellular matrix organization during adipogenesis (Griffin et al., 2002; Myneni et al., 2015). In these reactions, protein-bound glutamine residues act as acyl donors and lysine ε-amino groups, or other primary amines, act as acyl acceptors, generating Nε-(γ-glutamyl)lysine isopeptide bonds (Lorand and Conrad, 1984; Griffin et al., 2002). Thus, transglutaminase activity depends on the presence and accessibility of reactive glutamine and lysine residues in substrate proteins. Although substrate recognition is influenced by protein conformation and local structural context rather than by a single universal consensus motif, peptide-library and substrate-profiling approaches have shown that the sequence environment surrounding glutamine donor residues can strongly influence TG2 reactivity (Keresztessy et al., 2006; Hitomi et al., 2009; Damnjanović et al., 2022). Polyglutamine sequences can act as efficient transglutaminase substrates, and shorter glutamine-containing motifs may increase the local availability of potential reactive glutamine residues, although they do not by themselves establish a protein as a direct TG2 substrate (Kahlem et al., 1996; Keresztessy et al., 2006).

We therefore examined whether transglutaminase is affected by biotin availability in differentiating 3T3-F442A adipocytes. Under biotin-sufficient conditions, transglutaminase protein levels increased during adipogenesis, whereas this increase was reduced under biotin-deficient conditions ( Figure 1A ). Immunofluorescence analysis showed transglutaminase localization at or near lipid droplet surfaces, where it overlapped with perilipin in biotin-replete adipocytes ( Figure 1B ). In contrast, this organization was diminished in biotin-deficient cells, consistent with impaired lipid droplet formation.

To explore whether transglutaminase activity is functionally associated with lipid accumulation, differentiating cells were treated with cadaverine, a competitive amine substrate that inhibits transglutaminase-mediated protein crosslinking (Lorand and Conrad, 1984; Griffin et al., 2002). Cadaverine supplementation reduced malic enzyme activity ( Figure 1C ), an NADPH-generating enzyme that supports fatty acid synthesis during adipogenesis. Cytosolic NADP-dependent malic enzyme catalyzes the oxidative decarboxylation of malate to pyruvate with reduction of NADP⁺ to NADPH and requires divalent metal ions; it is not a classical biotin-dependent enzyme (Hsu, 1982; Chang and Tong, 2003). Therefore, the decrease in malic enzyme activity observed after cadaverine treatment is unlikely to reflect direct loss of a biotin prosthetic group on malic enzyme. Mouse cytosolic malic enzyme 1 contains multiple glutamine and lysine residues, including several short QQ motifs and one KK motif, but lacks an obvious extended glutamine- or lysine-rich repeat domain. Thus, although direct transglutaminase-mediated modification of malic enzyme remains possible, the primary sequence alone does not establish malic enzyme as a direct transglutaminase substrate. The decrease in malic enzyme activity after transglutaminase inhibition may therefore reflect either direct modification of malic enzyme or indirect effects on cytoskeletal organization, lipid droplet assembly, or the metabolic state of differentiating adipocytes. This reduction in malic enzyme activity was accompanied by decreased lipid accumulation, as detected by BODIPY staining ( Figure 1D ). Quantitative analysis of BODIPY fluorescence demonstrated significant differences in lipid accumulation among undifferentiated fibroblasts and adipocytes cultured under lipogenic, non-lipogenic, or transglutaminase inhibition (cadaverine) conditions. Statistical analysis was performed using one-way analysis of variance (ANOVA) followed by Bonferroni's multiple-comparisons post hoc test. Lipogenic differentiation resulted in significantly greater lipid accumulation than that observed in undifferentiated fibroblasts, non-lipogenic cultures, or cadaverine-treated cultures. Likewise, non-lipogenic and cadaverine-treated adipocytes differed significantly from lipogenic cultures and from undifferentiated fibroblasts. All pairwise comparisons were highly significant ( Figure 1E ), suggesting that both biotin deficiency and transglutaminase inhibition were associated with a marked reduction in lipid droplet accumulation during adipocyte differentiation.

Together, these findings identify transglutaminase as a biotin-sensitive component whose expression and localization are associated with lipid accumulation in 3T3-F442A adipocytes. The results extend earlier observations that biotin deficiency separates adipocyte differentiation from lipid storage, preserving induction of primary differentiation-associated markers such as glycerol-3-phosphate dehydrogenase while reducing secondary lipogenic activities such as malic enzyme (Kuri-Harcuch et al., 1978). The novel aspect of this study is the identification of transglutaminase as a biotin-sensitive factor that correlates with lipid droplet organization and lipogenic enzyme activity in differentiating 3T3-F442A white adipocytes. Although inhibition of transglutaminase activity was accompanied by reduced malic enzyme activity and lipid accumulation, these findings support an association between transglutaminase activity and adipocyte lipid storage rather than establishing a direct causal relationship.

One limitation of the present study is the reliance on cadaverine as a pharmacological inhibitor of transglutaminase activity. Although cadaverine is widely used as a competitive amine substrate to inhibit transglutaminase-mediated protein crosslinking, it is not specific for TG2 and pharmacological inhibition alone cannot exclude indirect or off-target effects (Lorand and Conrad, 1984; Griffin et al., 2002; Gundemir et al., 2012). In addition, pharmacological inhibition primarily interferes with the enzymatic transamidase activity of TG2 but does not eliminate the protein itself or distinguish between its catalytic and non-catalytic scaffold or signaling functions, which have been implicated in multiple cellular processes (Gundemir et al., 2012; Eckert et al., 2014 ). Conversely, genetic approaches such as siRNA-mediated knockdown or CRISPR-mediated deletion reduce TG2 expression and therefore can reveal functions that are independent of catalytic activity, although these strategies may also induce compensatory cellular responses or developmental adaptations that complicate interpretation. Thus, pharmacological inhibition and genetic manipulation provide complementary rather than interchangeable approaches for defining TG2 function. Future studies combining selective pharmacological inhibitors with loss- and gain-of-function genetic approaches will be important to determine the specific contribution of TG2 to lipid droplet organization and lipogenic metabolism in differentiating adipocytes.

Overall, the data support the hypothesis that biotin availability and transglutaminase activity are associated with the structural and metabolic remodeling that accompanies lipid droplet accumulation during adipocyte differentiation. Future studies combining pharmacological and genetic approaches will be necessary to establish the molecular mechanisms underlying this relationship and to determine whether transglutaminase directly regulates malic enzyme activity, lipid droplet-associated proteins, or cytoskeletal remodeling during adipogenesis.

## Methods

**Cell culture -** Bovine calf serum (BCS) and adult bovine serum were obtained from HyClone/Thermo Fisher Scientific (Waltham, MA). Dulbecco’s modified Eagle’s medium (DMEM) was purchased from Life Technologies (Carlsbad, CA). Epidermal growth factor (EGF) was obtained from EMD Millipore (Billerica, MA). Insulin, D-biotin, human transferrin, triiodothyronine (L-T3), and bovine serum albumin (BSA) were obtained from Sigma-Aldrich (St. Louis, MO). All other reagents were of analytical grade.

3T3-F442A preadipocytes were cultured as previously described (Green and Kehinde, 1976; Salazar-Olivo et al., 1995). Three days after plating, cells were induced to differentiate in adipogenic medium consisting of DMEM supplemented with 4% (v/v) BCS, insulin (5 µg/mL), and 1 µM D-biotin, and were refed every other day (Kuri-Harcuch and Green, 1978). After 48 h, cultures were switched to experimental adipogenic media: (i) lipogenic medium containing 4% (v/v) BCS, insulin (5 µg/mL), and 1 µM D-biotin; or (ii) non-lipogenic medium lacking added biotin, prepared with extensively dialyzed BCS and supplemented with insulin (5 µg/mL) (Kuri-Harcuch et al., 1978; Padilla-Benavides et al., 2016). Both conditions support adipogenic differentiation, but only the biotin-containing lipogenic medium permits robust lipid accumulation (Kuri-Harcuch et al., 1978; Padilla-Benavides et al., 2016). Cells were maintained at 37°C in a humidified atmosphere of 10% CO₂ and refed three times per week until fixation or extraction.

**Immunocytochemistry and confocal microscopy -** Cells were fixed at the indicated time points with 3.5% (w/v) paraformaldehyde in PBS for 2 h at 4°C, permeabilized with 0.05% Triton X-100 in PBS, and blocked with 10% (v/v) BCS in PBS for 2 h at room temperature (Blanchette-Mackie et al., 1995; DiDonato and Brasaemle, 2003; Padilla-Benavides et al., 2016). Primary antibodies against Plin1 (guinea pig; Research Diagnostics, Flanders, NJ) and transglutaminase (Abcam, ab421, Cambridge, UK) were applied for 1 h at 37°C. Secondary antibodies were Alexa Fluor 488-conjugated goat anti-guinea pig IgG and Cy5-conjugated goat anti-mouse IgG (Invitrogen, Carlsbad, CA).

Neutral lipid droplets were visualized with BODIPY 493/503 (4,4-difluoro-1,3,5,7,8-pentamethyl-4-bora-3a,4a-diaza-s-indacene; Molecular Probes, Eugene, OR). Cells were washed with PBS, fixed with 4% paraformaldehyde for 15-20 min, and incubated with 5 µM BODIPY 493/503 diluted in PBS or serum-free medium for 30 min at room temperature in the dark. Cells were then washed three times with PBS and imaged immediately by fluorescence or confocal microscopy using excitation/emission wavelengths of 493/503 nm.

Fluorescently labeled samples were mounted using Dako fluorescent mounting medium (Dako, Carpinteria, CA). Images were acquired using a Leica TCS SP2 confocal microscope (Leica Microsystems, Wetzlar, Germany) and processed using Leica Confocal Software version 2. Fluorescence intensity of lipid droplets stained with BODIPY 493/503 was quantified using ImageJ version 1.46 (Schindelin et al., 2012).

**Western blot analysis -** Cells were harvested at the indicated time points, washed with PBS, and lysed in buffer containing 10 mM PIPES, pH 7.4, 150 mM NaCl, 2 mM EDTA, 1% Triton X-100, 0.5% sodium deoxycholate, and 10% glycerol, supplemented with protease inhibitors (Roche Diagnostics, Indianapolis, IN). Protein concentration was determined by the Lowry method. Equal amounts of protein (10 µg) were resolved by SDS-PAGE and transferred to PVDF membranes (Millipore). Membranes were blocked for 2 h at room temperature in PBS containing 5% non-fat milk. Primary antibody incubation was performed for 1 h at room temperature using rabbit anti-transglutaminase at a 1:200 dilution in blocking solution. Membranes were washed six times for 5 min each with TBS containing 0.05% Tween-20 (TBS-T), followed by incubation with HRP-conjugated goat anti-rabbit IgG (ICN/Cappel, cat. 55689) at a 1:100 dilution for 1 h at room temperature. After additional washes with TBS-T, immunoreactive bands were detected using luminolbased chemiluminescence with a 1 min incubation in ECL Plus reagent (GE Healthcare Biosciences, Pittsburgh, PA). Membranes were stained with Ponceau S to verify transfer and used as loading controls (Romero-Calvo et al., 2010). Densitometric analysis was performed using ImageJ version 1.46 (Schindelin et al., 2012).

**Cell extracts and malic enzyme activity -** Culture medium was removed at the indicated time points, and cells were washed three times with PBS. Cell layers were scraped with a rubber policeman, resuspended in 0.2 mL of 0.25 M sucrose at 4°C, and disrupted by sonication. Lysates were centrifuged at 8,000 × g for 2 min to remove debris. The supernatant, containing approximately 85% of total protein in soluble or small particulate form, was carefully collected while avoiding the lipid layer. Samples of 0.1 mL were stored at -80°C until use. All procedures were performed at 5°C.

**Malic enzyme activity** - L-malate:NADP⁺ oxidoreductase (decarboxylating), EC 1.1.1.40, was measured at 30°C as described by Wise and Ball (1964), by monitoring NADP⁺ reduction at 340 nm using a continuously recording spectrophotometer. Protein concentration was determined by the method of Lowry et al. using bovine serum albumin as standard.

**Statistical analysis.** Data are presented as the mean ± standard error (SE) from three independent biological replicates. Statistical analyses were performed using KaleidaGraph software version 4.0.1 (Synergy Software). Pairwise comparisons between two matched experimental conditions were assessed using a paired, two-tailed Student’s t-test, including comparisons of biotin-sufficient (lipogenic) *vs.* biotin-deficient (non-lipogenic) cultures, lipogenic *vs.* cadaverine-treated cultures, and differentiated adipocytes *vs.* proliferating preadipocytes. Comparisons involving more than two groups were analyzed using one-way analysis of variance (ANOVA), followed by Bonferroni’s multiple-comparisons *post hoc* test. P values < 0.05 were considered statistically significant.
